# Peritoneal Dialysis Is an Independent Factor Associated to Lower Intima Media Thickness in Dialysis Patients Free From Previous Cardiovascular Disease

**DOI:** 10.3389/fphys.2018.01743

**Published:** 2018-12-04

**Authors:** Mercè Borràs, Serafí Cambray, Maria Crespo-Masip, Miguel Pérez-Fontán, Milica Bozic, Marcelino Bermudez-López, Elvira Fernández, Àngels Betriu, José M. Valdivielso

**Affiliations:** ^1^Vascular and Renal Translational Research Group, Biomedical Research Institute (IRBLleida), RedinRen RETIC, Instituto de Salud Carlos III, Lleida, Spain; ^2^Nephrology Department, University Hospital Arnau de Vilanova, Lleida, Spain; ^3^Nephrology Department, A Coruña Hospital, A Coruña, Spain

**Keywords:** intima and media thickness, dialysis, cohorts study, ultrasound, atherosclerosis

## Abstract

Carotid intima media thickness (cIMT) displays prognostic value as a marker of cardiovascular risk in dialysis patients. However, few data are available regarding the impact of dialysis modality on cIMT. The aim of this study is to determine whether the modality of dialysis influences cIMT values. We compared 237 peritoneal dialysis (PD) and 451 hemodialysis (HD) patients without previous cardiovascular disease included in NEFRONA, a prospective, observational and multicenter study. This cross sectional study included the determination of cIMT in 6 carotid territories by arterial ultrasound. cIMT was determined in territories without atheroma plaque and averaged. A second analysis was performed using all territories, giving a truncated cIMT value of 1,5 mm to territories presenting with atheroma plaque. Age and plaque presence at baseline were the clinical variables more closely associated to cIMT in dialysis patients. The evaluation of the impact of the modality of dialysis on cIMT showed that PD patients had lower cIMT than HD patients, both in territories with no plaques and when using truncated cIMT values. No differences were found between right and left sides, neither in cIMT or truncated cIMT values. Lineal multivariate analysis adjusted by several clinical variables showed a statistically significant association of PD with a lower cIMT (slope -0.036; SD 0.010). These results were also confirmed when truncated cIMT values were used. We conclude that the modality of dialysis has an impact on cITM. HD patients have greater global cIMT than PD patients, and PD is and independent factor associated with a lower cIMT.

## Introduction

In the last 35 years, the incidence of end-stage renal disease (ESRD) has raised dramatically. ESRD patients have a 5-year survival probability of 50% (United States Renal Data System (USRDS), 2017; Kramer et al., 2018) and, compared to general population, have a crude excess mortality rate of 80 to 175.(Foster et al., 2018) Main contributors to this increased mortality rates are dialysis patients, and among them, cardiovascular disease (CVD) accounts for 53% of the deaths.(United States Renal Data System (USRDS), 2017) In chronic kidney disease (CKD) patients, atherosclerosis hallmarks are more advanced than in the general population.(Betriu et al., 2014) This increase in atherosclerosis burden is parallel to the degree of kidney impairment, being higher in dialysis patients (Drueke and Massy, 2010). The contribution of atherosclerosis to the increase in mortality of ESRD patients has been questioned because most of the deaths are attributed to sudden death, and related to electrolyte imbalance. However, recent results have shown that atherosclerosis presence is an independent factor associated with cardiovascular events (CVE) in ESRD, and that each new arterial territory with plaque raised a 86% the hazards of having a CVE (Valdivielso et al., 2017).

Traditional risk factors are not useful to predict CVE on dialysis patients. The use of the traditional algorithms in risk stratification in CKD consistently underestimate the risk of the CKD patients of having a CVE (Coll et al., 2010). Therefore, new markers of cardiovascular risk in dialysis patients are needed. Among the candidates, vascular determination of intima media thickness of carotids (cIMT) has shown promising prognostic value in this population (Benedetto et al., 2001). Current knowledge about the impact of the type of dialysis on cIMT is based on studies in small cohorts, without adjustment for another possible confounding factors (Konings et al., 2002; Al-Hweish et al., 2010; Shi et al., 2012). These sparse data obtained in different cohorts, and with different methodology and criteria, precludes knowing whether peritoneal dialysis (PD) and hemodialysis (HD) affect cIMT differently.

There are significant differences between both modalities of dialysis. PD patients have a potentially higher atherogenic profile than their counterparts on HD, due to the recurrent peritoneal loading with glucose-based dialysis solutions, and to the continuous peritoneal leak of proteins. On the contrary, HD patients show a worse preservation of residual kidney function, which may contribute to inflammation, endothelial dysfunction and vascular calcification. Moreover, PD is associated with a stable fluid status and blood pressure pattern compared to periodic fluctuations found in HD. Therefore, the modality of dialysis itself could have a differential effect on cIMT determining factors.

With the aim of shedding some light on this clinical concern, we evaluated cIMT in a selected sub-cohort of the NEFRONA study, in order to determine whether the modality of dialysis influences cIMT.

## Materials and Methods

### Study Design and Participants

The ethics committee of University Hospital Arnau de Vilanova from Lleida, Spain, approved the study. All included patients signed informed consent and the study complied with the principles of the Declaration of Helsinki.

The NEFRONA project is a prospective, multicentre, observational cohorts study from Spain aimed to assess the atherosclerotic burden in CKD patients, including patients with ESRD. The rationale and baseline description of NEFRONA cohort have been reported in detail elsewhere. (Junyent et al., 2010a,b) Briefly, 2445 CKD patients free from previous CVE, aged 18 to 75 were enrolled from 81 Spanish hospitals between October 2009 and June 2011. The exclusion criteria were previous CVE, active infections (HIV, tuberculosis), pregnancy, having received any organ transplantation or having a life expectancy of less than 1 year. This study is a cross-sectional analysis in a subcohort of the NEFRONA study, including all the dialysis patients recruited (451 on HD and 237 on PD).

### Clinical and Biochemical Data

At recruitment, patients were asked to complete a questionnaire including family history regarding premature CVD, clinical history (diabetes, hypertension and dyslipidaemia), cardiovascular risk factors (such as smoking habit), and medication use. Anthropometric data and medical history were also obtained from all patients at the time of recruitment. Biochemical data were obtained from a routine blood test 3 months either before or after the vascular ultrasound. For HD patients, blood samples were retrieved just before the second session of the week. High-sensitivity C reactive protein (hsCRP), 25-hydroxy-vitamin D and 1,25-hydroxy-vitamin D were quantified in a centralized laboratory. ABI measurements were performed with a protocol previously described (Arroyo et al., 2017).

### Carotid Ultrasound

Patients underwent B-mode ultrasound in both carotid arteries with the Vivid BT09 device (General Electric Instruments, Freiburg, Germany) and a 6–13 MHz broadband linear array probe. For imaging, patients were in supine position with the head turned 45° contralateral to the side of the probe. cIMT was measured in the last centimeter of the far wall of the common carotid artery, the bulb section and in the first centimeter of the internal carotid artery. Measurements were made in plaque-free arterial segments. Atheromatous plaque, following the recommendations of the ASE Consensus Statement (Stein et al., 2008) and the Mannheim cIMT Consensus report (Touboul et al., 2004), was defined by a cIMT ≥ 1.5 mm protruding to the lumen of the imaged sections. In order to account for the values of cIMT in territories with plaques, a truncated cIMT value was also calculated. Truncated cIMT calculations were made giving a cIMT value equal to 1.5 mm to the territories with plaque. In each patient, averaged cIMT true values or truncated values of the six territories explored were calculated.

Ultrasound explorations were carried out by the same itinerant team of five trained technicians. Images were analyzed by a single reader in a blinded fashion using the EchoPAC Dimension software (General Electric Healthcare, Harten, Norway) in the UDETMA (Unit for the Detection and Treatment of Atherothrombotic Diseases, Hospital Universitari Arnau de Vilanova, Lleida, Spain). To assess the quality of the measurements a sample of 20 individuals was measured 3–5 times on different days, obtaining an intraclass correlation coefficient of 0.93.

### Statistical Analysis

Quantitative variables are shown as means and standard deviations, and its differences between groups were compared with the Student’s *t*-test. Qualitative variables are summarized as absolute and relative frequencies, and chi-squared test (Fisher test for expected frequencies <5) was used to perform comparisons between groups. Pearson′s correlations were used to determine univariate relationships between cIMT values and linear or categorical variables. Significant variables in univariate analyses and potential confounders were used to develop appropriate multivariate linear regression models. A forward step procedure was used to build the multivariate model, including the variables showing maximum contribution identifying those patients with higher cIMT, according to the likelihood ratio test (LRT).

## Results

### Baseline Characteristics

A total of 451 HD and 237 PD patients were included. Table 1 shows anthropometrical, clinical and biochemical data comparisons between both groups. PD patients were younger, showed increased ratios of hypertension and dyslipidemia, and had higher systolic arterial pressure, total cholesterol, HDL and LDL cholesterol, haematocrit, calcium and phosphate; they had been less time on dialysis and presented with lower levels of potassium and 25-hydroxy-vitamin D.

**Table 1:** Demographic data.

	HD (*n* = 451)	PD (*n* = 237)	*p*
Age (years)	54 (14)	51 (14)	0,004
Sex (man)	272 (60,3)	137 (57,8)	0,567
Smoker	250 (55,4)	135 (57,0)	0,381
Diabetes	107 (23,7)	47 (19,8)	0,143
Hypertension	406 (90,0)	229 (96,6)	0,001
Dyslipidemia	214 (47,5)	153 (64,6)	0,000
Plaque presence at baseline	325 (72,1)	156 (65,8)	0,054
BMI (kg/m^2^)	26,3 (5,0)	26,7 (4,8)	0,341
SBP (mmHg)	136 (23)	144 (24)	0,000
Time on dialysis (months)	33 (43)	20 (19)	0,000
Total cholesterol (mg/dL)	156 (39)	180 (43)	0,000
HDL cholesterol (mg/dL)	46 (16)	49 (15)	0,016
LDL cholesterol (mg/dL)	84 (32)	104 (34)	0,000
Triglycerides (mg/dL)	141 (81)	137 (66)	0,498
Glucose (mg/dL)	103 (40)	101 (37)	0,526
Hematocrite (%)	35,7 (4,3)	36,6 (4,4)	0,013
Calcium (mg/dL)	9,0 (0,7)	9,2 (0,7)	0,005
Phosphate (mg/dL)	4,8 (1,4)	5,1 (1,2)	0,008
Uric acid (mg/dL)	6,1 (1,4)	6,0 (1,2)	0,22
iPTH (pg/mL)	309 (281)	277 (255)	0,164
Sodium (mEq/L)	139 (3)	139 (3)	0,036
Potassium (mEq/L)	5,1 (0,8)	4,5 (0,6)	0,000
usCRP (mg/L)	5,6 (10,7)	6,1 (13,9)	0,624
25OH vitamin D (ng/mL)	16,4 (8,3)	12,8 (5,5)	0,000
1,25(OH)_2_ vitamin D (pg/mL)	8,6 (5,1)	7,7 (4,8)	0,039
Treatment with Calcium-containing P binders	186 (41,2)	119 (50,2)	0.015
Total Kt/V	1,56 (0,39)	2,52 (0,63)^∗^	NC
Calcium content in dialysis fluid			0.001
2.5 mEq/L	182 (41,9)	72 (35.8)	
3 mEq/L	221 (50,9)	NA	
3,5 mEq/L	31 (7,1)	129 (64.2)	

*Quantitative data are shown as means (SD). Qualitative data are shown as number (%). HD, Hemodialysis; PD, Peritoneal Dialysis; BMI, Body mass index; SBP, Systolic blood pressure; iPTH, Intact parathyroid hormone; usCRP, Ultra-sensitive C reactive protein; NA, Not available. The concentration of 3 mEq/L is not available for PD dialysis fluid. NC, non comparable; ^∗^weekly Kt/V.*

Variables influencing cIMT on dialysis patients.

**FIGURE 1:**
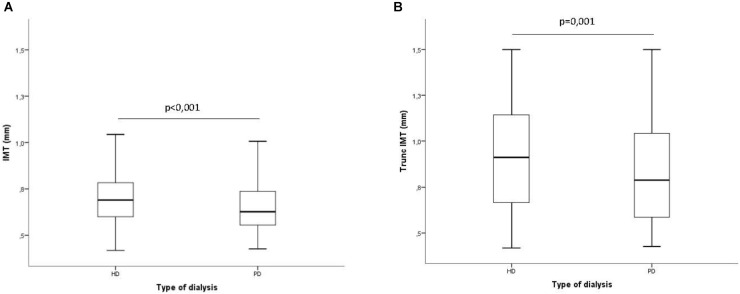
Averaged intima media thickness in carotid arterial territories depending on the dialysis modality. **(A)** Excluding territories with atheroma plaque; **(B)** Including territories with atheroma plaque. In territories with plaque, the IMT value was truncated to 1,5 mm.

Figure 1 shows averaged cIMT values in HD and PD patients both in territories with no plaques (IMT) and in territories in which the values of cIMT were truncated to 1.5 mm when there was a plaque present (truncated cIMT). In both cases, PD patients showed lower levels of cIMT than HD patients.

Among all clinical and biochemical variables considered, the ones more closely related to cIMT were age and plaque presence at baseline, both positively correlated (Table 2). The other variables with positive correlation with cIMT were body mass index (BMI), diabetes, dyslipidemia, ankle-brachial index (ABI), potassium and glucose. The only variable with negative correlation to cIMT was phosphate levels. We also found that being male and in HD were correlated with a higher cIMT. When truncated cIMT was considered, all the correlations and significances were exacerbated except the one with phosphate, which became non-significant.

**Table 2:** Statistically significant correlations between different parameters and cIMT values.

	IMT		IMT truncated	
	*R*	*p*	*r*	*p*
Sex (women)	−0.138	0.000	−0.137	0.000
Age	0.545	0.000	0.613	0.000
BMI	0.232	0.000	0.233	0.000
Diabetes	0.213	0.000	0.271	0.000
Dyslipidemia	0.077	0.049	0.129	0.001
ABI	0.082	0.037	0.137	0.000
Phosphate	−0.086	0.027		
Potassium	0.112	0.004	0.130	0.001
Glucose	0.122	0.002	0.158	0.000
Plaque presence at baseline	0.421	0.000	0.663	0.000
Type of dialysis (PD)	−0.165	0.000	−0.130	0.001

**FIGURE 2:**
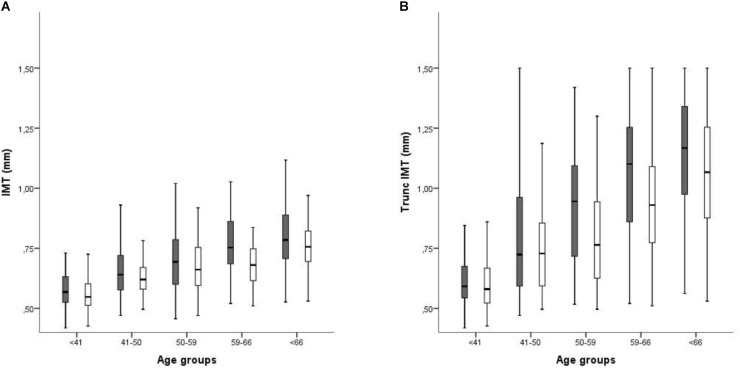
Averaged intima media thickness in carotid arterial territories stratified by age and sex. Dark bars: Males; White bars: Females. **(A)** Excluding territories with atheroma plaque; **(B)** Including territories with atheroma plaque. In territories with plaque, the IMT value was truncated to 1,5 mm. *p* trend > 0,01.

**FIGURE 3:**
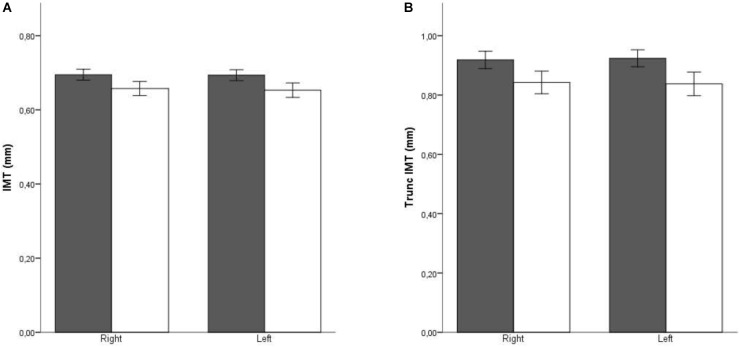
Averaged intima media thickness in carotid arterial territories stratified by side. Dark bars: HD patients; White bars: PD patients. **(A)** Excluding territories with atheroma plaque; **(B)** Including territories with atheroma plaque. In territories with plaque, the IMT value was truncated to 1,5 mm.

When patients were stratified according to sex and age, the relationship of these variables to cIMT was clearly visible, with cIMT values increasing with age (showing a statistically significant *p*-value for the trend), and more abruptly in men (both in normal cIMT and truncated cIMT) (Figure 2). Furthermore, the difference related to sex was maintained in all age groups. The differences in laterality are also depicted in Figure 3. No differences in cIMT were observed between left and right carotids, being always higher in HD patients, regardless of the side or whether we analyzed cIMT or truncated cIMT values.

To better assess the influence of dialysis type on cIMT, we generated different models by means of lineal multivariate analysis. Univariate regression assessing the effect of dialysis type on cIMT showed a statistically significant association of PD with a lower cIMT value. (Table 3). After adjusting by sex and age (Model 2) the association between cIMT and type of dialysis remained significant. This association was maintained even after adjusting by several clinical variables in model 3 (Diabetes, Dyslipidemia, Hypertension, BMI, ABI, Plaque presence at baseline, Total Cholesterol, LDL Cholesterol, Glucose, Potassium, SBP, time on dialysis) confirming an association of PD with lower cIMT values. When truncated cIMT values were used as the dependent variable, the effect of dialysis type on cIMT remained. Only statistically significant variables were maintained in the final model and shown in the table.

**Table 3:** Lineal multivariate analysis.

	Model 1			Model 2			Model 3		
	Slope	*SD*	*p*	Slope	*SD*	*p*	Slope	*SD*	*p*
**cIMT**									
Type of dialysis (DP vs. HD)	−0.051	0.012	0.000	−0.034	0.010	0.001	−0.033	0.010	0.001
Sex (women vs. men)				−0.041	0.010	0.000	−0.036	0.010	0.000
Age (year)				0.013	0.001	0.000	0.004	0.000	0.000
Hypertension							0.041	0.017	0.016
Plaque presence at baseline							0.036	0.012	0.002
Diabetes							0.033	0.012	0.006
**Truncated cIMT**									
Type of dialysis (DP vs. HD)	−0.084	0.025	0.001	−0.041	0.019	0.033	−0.044	0.017	0.008
Sex (women vs. men)				−0.076	0.019	0.000	−0.038	0.017	0.023
Age (year)				0.005	0.000	0.000	0.008	0.001	0.000
BMI							0.004	0.002	0.019
SBP (mmHg)							0.001	0.000	0.005
Plaque presence at baseline							0.269	0.021	0.000
Diabetes							0.063	0.020	0.002

*Model 1 unadjusted. Model 2 adjusted for sex and age. Model 3 adjusted by sex, age, diabetes, dyslipidemia, hypertension, BMI, ABI, plaque presence at baseline, Total Cholesterol, LDL Cholesterol, Glucose, Potassium, SBP, time on dialysis, treatment with calcium-containing phosphate binders, calcium levels in dialysis fluid.*

## Discussion

Our results show that on dialysis patients, cIMT values correlate to gender, age, plaque presence at baseline, BMI and diabetes. In non-dialysis population, previous studies also found association of cIMT with age and sex (Dobs et al., 1999; Mutluay et al., 2012), plaque presence (Ebrahim et al., 1999), BMI (Berni et al., 2011) and diabetes (Brohall et al., 2006). Furthermore, our results show that the modality of dialysis has an impact on cIMT, even when adjusting by possible confounders. Thus, HD patients have greater global cIMT than PD patients and being in PD is independently associated with a lower cIMT.

Currently, studies about dialysis impact on ultrasound measured cIMT have shown contradictory results. Despite all studies have found higher cIMT on dialysis patients than in non-dialysis population,(Cengiz and Dolu, 2007; Mutluay et al., 2012; Shi et al., 2012) some of them reported higher cIMT in PD than in HD (Ozdemir et al., 2001; Shi et al., 2012), some other did not find significant differences on cIMT between dialysis type (Cengiz and Dolu, 2007; Yilmaz et al., 2007; Al-Hweish et al., 2010), and some others reported increased cIMT in HD patients (Mutluay et al., 2012). However, it is important to remark that, in contrast to most previous studies, the NEFRONA study excluded patients with a previous CVE. Indeed, the only similar study that also excluded patients that suffered a CVE (although with a quite lower number of patients that our study) also found that HD dialysis patient have higher cIMT (Tonbul et al., 2006).

Recently, the same NEFRONA cohort with a matched case-control design revealed that the modality of dialysis did not influence atheromatous vascular disease progression (assessed as an increase in the number of atheromatous plaques) neither did cardiovascular outcomes (Borràs Sans et al., 2017). These results suggest that plaque and cIMT may be distinct phenotypes rather than a manifestation of the same phenotype at different stages or phases in the progression of atherosclerosis, being in line with recent studies. Thus, the Northern Manhattan Study showed that the association between elevated baseline cIMT and the risk of new plaque formation disappeared after adjusting for demographic and vascular risk factors (Rundek et al., 2015). Similarly, Baroncini et al. (2015) found that increased cIMT was related to hypertension, but plaque presence was associated to age and dyslipidemia. Therefore, the results seems to show that despite increased cIMT and atherosclerosis are commonly found together, the risk factors influencing one or another could be different and may be genetically and biologically distinct atherosclerotic phenotypes with a heterogeneous etiology (Spence, 2006; Della-Morte et al., 2012). Therefore, plaque may not be a simple result of progressive intima-media thickening, but rather a new and different event. Indeed the hypothesis that increases in cIMT might be an adaptive event of the median layer to increased shear stress, rather than an atherosclerotic sign, is gaining adepts nowadays.

The possible causes of higher cIMT in HD patients than in PD patients are unknown. However, it is well stablished that hypertension, and particularly blood pressure variability (BPV), plays a major role in cIMT increase (Mancia et al., 2001; García-García et al., 2013). Short and long-term BPV are inherent to HD treatment because of day-to-day fluctuations of volume status. Moreover, cIMT in HD patients is more associated with long (24- and 48-h ambulatory BP measurements) than routine dialysis center BP measurements (Ekart et al., 2009). On the contrary, patients in PD have more volume status stability and, accordingly, they should present less BPV. Indeed, HD is associated with higher systolic BPs and greater systolic loads than PD in a study monitoring BP by continuous ambulatory blood pressure measurements (Rodby et al., 1994). One may hypothesize that the difference in short and long-term BPV between HD and PD patients could explain the higher cIMT in HD patients despite having lower casual BP measurements. In the same line of reasoning, Günal et al. (2006) showed a decrease in cIMT with an strict volume control in HD patients, a fact that has not been proven to be true in PD patients (Hiramatsu et al., 2007). As a last hypothesis for the increased cIMT in HD patients, there is the possibility that the previously described increased levels of pro-BNP in HD patients (Nakatani et al., 2002; Al-Hweish et al., 2010; Sanjuan et al., 2011) could favor an increased cIMT, as it has been published for CKD patients (DeFilippi et al., 2005; Sathi et al., 2014); a similar effect has been described for Troponins (Al-Hweish et al., 2010; Caliskan et al., 2012).

There is growing evidence that calcium supplements can increase atherosclerotic CVE (Bolland et al., 2008, 2010; Li et al., 2012; Mao et al., 2013), although some reports have shown no statistically significant effect (Wang et al., 2010; Lewis et al., 2011). Therefore, there is a concern that calcium supplements can increase atherosclerosis. Dialysis patients can have a significant calcium intake, mainly due to the calcium content in the dialysis fluid and in phosphate binders administered to control hyperphosphatemia. In our study, there was a statistically significant difference in calcium supplements, as a higher percentage of PD patients received calcium-containing phosphate binders and were treated with dialysis fluid containing higher concentrations of calcium. This can be the cause of the small but significant increase in serum calcium levels. However, and although an inverse relationship between calcium load and IMT is suggested in the bivariate analysis, the difference did not reach statistical significance after adjustment for potential confounders.

Another risk factor for atherosclerosis development is hypertension (Hurtubise et al., 2016). Again, the percentage of patients diagnosed with hypertension and the SBP levels are higher in patients in PD, precisely those with lower IMT values. The multivariate analysis demonstrated that the statistical significance for both parameters was lost after adjustment. Therefore, either the effect of HD on IMT is so strong that can overcome the effect of common risk factors like hypertension, or the increases in IMT are influenced by different risk factors than those affecting atheroma plaque formation. Interestingly, PD patients were more dyslipidemic and presented with higher serum LDL-cholesterol levels than those in HD. This could be considered not only as one more piece of evidence supporting the hypothesis of increases in cIMT beyond an atherosclerotic sign or a consequence of some protective effect of PD versus HD, but also as another example of the controversial significance of LDL-cholesterol levels in dialysis patients.

Our results also show that there are no differences between left and right sides in cIMT neither, in PD nor HD patients. Several reports have shown a significantly higher proportion of cerebral ischemic events diagnosed in the left hemisphere than in the right (Hedna et al., 2013). An explanation for the higher incidence of events in the left hemisphere may be related to a higher prevalence, severity, or vulnerability of atherosclerotic disease in the left carotid artery. This is primarily attributable to differences in flow velocity in the left carotid artery, resulting in higher stress and intimal damage (Rodríguez Hernández et al., 2003). However, recent reports show that the higher incidence in left hemisphere strokes might be due to an increase in the vulnerability of the plaques, rather than in plaque formation (Adams et al., 2002; Selwaness et al., 2014). Thus, in our study, no differences in cIMT were found between right and left sides, suggesting that a systemic factor is involved in the increased cIMT found in HD patients.

Strengths of our study are the relatively high number of patients being, as far as we know, the biggest cohort used to determine the effects of dialysis on cIMT. Therefore, we have been able to adjust the models for many potential confounders. Furthermore, and although the study is multicentric, the execution and the analysis of the ultrasounds have been made by a single team, avoiding the high variability associated to the cIMT measurements. On the other hand, we have to mention some limitations. First, the exclusion of patients that presented CVE on the recruitment phase avoid extrapolating our results to the entire ESRD population. Second, we only have a single BP measurement and no fluid status data that could support the hypothesis that higher BPV in HD patients may be related to thicker cIMT. Third, the lack of determinations of cardiovascular biomarkers that could affect cIMT like pro-BNP or troponins, preclude us to determine whether the effect of dialysis modality is related to these biomarkers or to some other factor.

In summary, we have shown that cIMT is higher in HD than in PD and that being in PD is an independent risk factor associated with lower cIMT in dialysis patients.

## Author Contributions

MeB, EF, ÀB, and JV designed the study. SC, MP-F, and JV analyzed the data. MC-M, MiB, and MB-L performed the experiments. JV wrote the manuscript.

## Conflict of Interest Statement

The authors declare that the research was conducted in the absence of any commercial or financial relationships that could be construed as a potential conflict of interest.
